# Racial/Ethnic Variances in COVID-19 Inoculation among Southern California Healthcare Workers

**DOI:** 10.3390/vaccines10081331

**Published:** 2022-08-17

**Authors:** Alex Dubov, Brian J. Distelberg, Jacinda C. Abdul-Mutakabbir, Bridgette Peteet, Lisa Roberts, Susanne B. Montgomery, Nicholas Rockwood, Pranjal Patel, Steven Shoptaw, Ara A. Chrissian

**Affiliations:** 1 School of Behavioral Health, Loma Linda University, Loma Linda, CA 92350, USA; 2 School of Pharmacy, Loma Linda University, Loma Linda, CA 92350, USA; 3 School of Nursing, Loma Linda University, Loma Linda, CA 92350, USA; 4 School of Medicine, Loma Linda University, Loma Linda, CA 92350, USA; 5 Department of Family Medicine, University of California Los Angeles, Los Angeles, CA 90032, USA

**Keywords:** COVID-19 pandemic, vaccine hesitancy, healthcare professionals, racial/ethnic groups, booster, COVID-19 knowledge, vaccine acceptance, targeted interventions

## Abstract

Healthcare workers (HCWs) from minoritized communities are a critical partner in moving vaccine-hesitant populations toward vaccination, yet a significant number of these HCWs are delaying or deciding against their own COVID-19 vaccinations. Our study aims to provide a more nuanced understanding of vaccine hesitancy among racially and ethnically minoritized HCWs and to describe factors associated with vaccine non-acceptance. Analysis of a sub-sample of racially and ethnically minoritized HCWs (N = 1131), who participated in a cross-sectional study at two large Southern California medical centers, was conducted. Participants completed an online survey consisting of demographics, work setting and clinical role, influenza vaccination history, COVID-19 knowledge, beliefs, personal COVID-19 exposure, diagnosis, and impact on those closest to them. While overall most HCWs were vaccinated (84%), 28% of Black, 19% of Hispanic, and 8% of Asian American HCWs were vaccine-hesitant. Age, education level, occupation, history of COVID-19, and COVID-19 related knowledge were predictive of vaccine hesitancy. We found significant variations in COVID-19 related knowledge and reasons for vaccine hesitancy among Black (governmental mistrust), Hispanic (preference for physiological immunity), and Asian-American HCWs (concern about side effects) who were vaccine-hesitant or not. Our findings highlight racial and ethnic differences in vaccine-hesitancy and barriers to vaccination among HCWs of color. This study indicates the necessity of targeted interventions to reduce vaccine hesitancy that are mindful of the disparities in knowledge and access and differences between and among racial and ethnic groups.

## 1. Introduction

In March 2020, coronavirus (COVID-19) disease was declared a global pandemic [1]. Over 6.1 million COVID-19-related deaths were recorded worldwide within two years, with over 974,000 deaths occurring in the U.S. and 87,702 in California alone [2]. This pandemic highlighted longstanding health disparities, specifically those causing disproportionate harm to historically marginalized groups. The Centers for Disease Control (CDC) state that in the U.S., racially and ethnically minoritized individuals (hereafter minoritized; e.g., Hispanic, African American, Native Americans) are 1.6 times more likely to be infected, 2.5 times more likely to be hospitalized, and 2.1 times more likely to die of COVID-19 than Whites [3]. 

With the approval of three vaccines that have targeted action against the SARS-CoV-2 pathogen, including two mRNA vaccines (Moderna and Pfizer) and a viral-vectored vaccine (Johnson and Johnson; Janssen), we are well-positioned to overcome the pandemic. Healthcare workers (HCWs) were the first group in the U.S. to be offered COVID-19 vaccinations. However, approximately 22.5% of HCWs worldwide were COVID-19 vaccine-hesitant [4]. The World Health Organization (WHO) defines vaccine hesitancy as refusing vaccination despite availability and accessibility [5]. 

Public confidence in vaccines has been decreasing for the past several years, leading the WHO to declare vaccine hesitancy as one of the ten most important issues in global health [6]. As such, reviews of vaccine hesitancy among HCWs [7,8] related mainly to influenza viruses, with an estimated prevalence of vaccine hesitancy among HCWs from 3% to 44%. While reasons for vaccine hesitancy vary between individuals and by context, the WHO’s ‘three C’s model’ [9] has identified three factors influencing vaccine hesitancy: convenience (vaccine access), confidence (trust in vaccines), and complacency (perceived risk of vaccine-related disease). Several validated measures of vaccine hesitancy [10,11] used the ‘three C’s model’ to assess vaccine decision-making. 

The impact of systemic racism, the history of research abuses among communities of color in the U.S., and the lived experience of mistreatment in the healthcare system have amplified vaccine hesitancy among minoritized groups [12]. This hesitancy is also present amongst minoritized healthcare workers (HCWs), irrespective of their proximity to this virus [13]. According to a 2021 study of 10,871 HCWs, vaccine hesitancy was nearly 5-fold higher among Black HCWs and 2-fold higher among Hispanic/Latino HCWs compared to their non-Hispanic White counterparts. Vaccination rates have been declining steadily since April 2021 [14]. 

HCWs’ trust or distrust of the COVID-19 vaccines affects patients’ willingness to get immunized. Since HCWs are responsible for providing patients with information about the benefits and risks of vaccinations, their vaccine hesitancy may exacerbate patient hesitancy, especially among minoritized groups [15]. These findings point to the importance of understanding the multiple and interconnected reasons underlying vaccine hesitancy among HCWs from minoritized communities, which would in turn lead to tailored and more effective education campaigns. Since there continues to be a shortage of research examining racial/ethnic differences in vaccine hesitancy and specific factors hindering vaccine uptake among communities of color [16].

This study aims to understand the prevalence and factors associated with vaccine hesitancy among HCWs from minoritized communities. A better understanding of vaccine hesitancy among HCWs of color is essential as recent vaccination mandates have minimal influence on vaccine hesitancy, leaving hospital administrators in need to find and evaluate tailored interventions to increase vaccination uptake [17]. To this, the study was guided by several assumptions: (1) most HCWs of color will accept vaccination, (2) vaccine hesitancy will vary by race/ethnicity, (3) predictors of vaccine hesitancy (including vaccine-related knowledge) and reasons for vaccine hesitancy will vary by race/ethnicity.

## 2. Materials and Methods

We conducted an online, cross-sectional survey of HCWs in two large hospital systems (academic and private) in Southern California. The study protocol was reviewed by the respective IRBs of each participating institution and deemed exempt as non-human subjects research. Both hospitals began vaccinating their HCWs against COVID-19 on 17 December 2020.

### 2.1. Sample and Recruitment Strategy

Recruitment occurred relatively early after vaccination distribution, between 5–26 February 2021 at the academic hospital and 3–17 April 2021, at the private hospital. We distributed the online Qualtrics survey via institution-wide email listservs, with 8848 recipients at the academic hospital and 3062 recipients at the private hospital. Listserv recipients included physicians, nurses, advanced practice providers, pharmacists, allied healthcare professionals, administrators, and non-clinical ancillary staff. All employees of both hospital systems were invited to participate in the study. The initial invitation email to complete the survey was followed by two email reminders to encourage participation. The survey was anonymous and voluntary. A total of 11,910 nurses, advanced practice providers, physicians, pharmacists, allied health professionals, administrators, and ancillary staff were sent the survey link, to which 2491 responded (20.9% response rate). Among the 2491 HCWs who responded to the survey, 123 identified as Black, 438 identified as Asian Americans, and 570 identified as Hispanic/Latino. The present study is an analysis of these 1131 HCWs of color.

### 2.2. Data Collection Process

A working group developed a survey to understand HCWs’ knowledge, attitudes, and perceptions about the COVID-19 vaccination. The survey was based on previous questionnaires conducted during the 2009 H1N1 flu pandemic. It was pilot-tested with seven healthcare professionals and revised to ensure readability and understandability. The final survey included forced-choice questions exclusively to avoid missing data.

### 2.3. Measures

The survey instrument was composed of five parts: (1) Demographics: including age, gender, race, ethnicity, education level, self-reported history of chronic illness, income level, household size, and political party affiliation; (2) Clinical characteristics, such as position within the healthcare field, clinical work setting, medical specialty, frequency of contact with COVID-19 patients, and self-reported history of flu vaccination; (3) COVID-19-related misinformation: belief in a synthetic origin of the virus, belief that it’s a hoax, and belief that its impact on the healthcare system is exaggerated; (4) COVID-19 knowledge: understanding that it is more deadly and contagious than seasonal flu, estimated mortality for self and an average American, and understanding vaccine effectiveness; and (5) COVID-19’s impact: financial impact and whether someone close to the respondent had a severe illness or death. Additionally, participants were asked directly about reasons for vaccine hesitancy and instructed to select all that applied. If they felt that the survey did not capture all their reasons in the statement items provided for vaccine hesitancy, they could choose ‘other’ and input text.

We derived the primary outcome, COVID-19 vaccine behavior/hesitancy, from two questions—(a) receipt of any dose of a COVID-19 vaccine and (b) in case of a negative answer, intention to receive a COVID-19 vaccine within the next six months. We considered a “no” to (a) above and subsequent response of “unsure”, “probably not”, and “definitely not” to (b) above indicative of COVID-19 vaccine hesitancy. The 37-item survey took, on average, 15 min to complete.

### 2.4. Analysis

We exported all quantitative data into SPSS 28.0 for analysis. We exported textual data for vaccine hesitancy to NVivo 12.0 for qualitative analysis. The research team first reviewed data at a univariate and descriptive statistics level and the ability of the data to conform to the assumptions of the planned analysis. An ‘I don’t know’ option was included as a response choice and, therefore, assessed as missing data. Overall, there was less than 1% missing data on all items except the ‘household income’ (7.4%) and the ‘perceived likelihood of dying from COVID-19’ items (21.5%). Before analysis, a Full Information Maximum Likelihood Imputation was applied to impute the missing values after items were determined to be missing at random. 

The outcome variable for the participant’s vaccination status was then assessed using logistic regression. The model attempted to fit the three-category outcome variable and yielded inadequate power due to the limited number of data points with an outcome of hesitant and no vaccination intention. Therefore, the final models fit the binary outcome of vaccinated versus vaccine-hesitant (non-vaccinated). First, all demographic variables were force-entered into the model. Participants reporting ‘white’ as their ethnicity were used as the reference group. Therefore, the full sample of *n* = 2491 were included in the model. Then, the remaining variables were fit to the model through backward stepwise removal. A stepwise process was employed to assure that the final model achieved adequate power. This first model identified multiple significant independent variables, and therefore a second model was fit to optimize power. This second model carried forward the force-entered demographic variables and kept all significant independent variables from the backward step removal modeling; we also added interaction terms with race/ethnicity and all other variables in the model. The models were run with the imputed data and missing data. No significant differences in outcomes or coefficients were noted. Imputed data results are presented in the Results section. 

Additionally, we conducted Chi-square tests to determine significant differences within and between the three racial/ethnic subgroups (Black, Asian American, and Hispanic) included in this study.

## 3. Results

### 3.1. Racial/Ethnic Differences in Demographic Characteristics

Table 1 presents the descriptive characteristics of the sample. Most of the 1131 participants of color identified as women (80%), were college-educated or higher (68%), and were most often in the 25–40-year-old range (48%). Nursing was the most represented occupation (34%), followed by other allied health professionals (32%), physicians (17%), and administrators (7%). Participants worked in various clinical settings, but most had no contact (38%) or limited contact (32%) with COVID-19 patients. The majority (85%) of the sample had no prior history of COVID-19. However, forty-six percent of participants stated that the pandemic had negatively affected them financially. Almost half of the sample (49%) identified as Democrat or Democrat-leaning, one-third (31%) reported no lean, and 20% were Republican or Republican-leaning. The vast majority (91%) were recently immunized against seasonal flu. Fifteen percent reported a prior diagnosis with COVID-19, and 47% said that someone close to them had suffered severe disability or died. 

There were several demographic differences among racial/ethnic groups represented in this study. A total of 1 in 4 Asian Americans (24%) had a household member over 65; 1 in 5 Black individuals (20%) had not received a seasonal flu vaccination; almost half (49%) of Hispanic participants had less than a bachelor’s degree and tended to work in ancillary services (43%) and outpatient areas (39%); and 1 in 5 (20%) Hispanic participants had been previously diagnosed with COVID-19. 

Most relevant to this paper, 28% of Black participants were vaccine-hesitant vs. 18.9% of Hispanics and 8% of Asian Americans. Across all three minoritized groups, vaccine-hesitant individuals were more Republican-leaning or, for Hispanic and Black participants, reported ‘no lean’. Additionally, among all three groups, they were more likely to have a prior COVID diagnosis and have had COVID in their families, though they were less likely to have someone die of COVID than those in their ethnic groups who were vaccinated.

### 3.2. Racial/Ethnic Differences in COVID Knowledge

The diversity of beliefs about the COVID-19 virus and the vaccine is shown in Table 2. A large majority of the sample (75%) correctly identified vaccine efficacy to be 90% or greater. At the time of the survey, the estimated COVID-19 Case Fatality Rate (CFR) was 0.5–1.5%. A significant proportion of respondents underestimated COVID-19 mortality risk (e.g., selecting responses with less than 0.5% risk) or overestimated risks (e.g., selecting responses with excessive risk levels of 3% and higher). We used the same approach (CFR) in coding estimates of self-risk. As such, over one-third (37%) of the sample believed that seasonal flu could be deadlier than COVID-19 and the same percentage (37%) of respondents overestimated mortality associated with COVID-19. One-third (34%) of the sample considered their risk of dying from COVID-19 as low, and 22% placed themselves at high risk for dying from COVID-19.

The prevalence of COVID-19 misinformation was measured by asking respondents to indicate their level of agreement with statements, such as “COVID-19 is a hoax”, using a 5-point Likert scale. We considered responses ‘unsure’, ‘somewhat agree’, and ‘definitely agree’ as indicative of entertaining COVID-19 misinformation observed among the non-trivial part of the sample. For instance, 41% of the sample believed the COVID-19 is or could be manufactured, 17% considered the impact of COVID-19 to be exaggerated, and 8% entertained the belief that COVID-19 could be a “hoax”.

While the prevalence of misinformation about COVID-19 and the vaccine was high, Asian Americans were less likely to be impacted by it. Yet, one in five (19%) Hispanic participants believed that the impact of COVID-19 on the healthcare system is or could be exaggerated. One-third (35%) of Black individuals had low confidence in the COVID-19 vaccine. Hispanic respondents were also more likely to underestimate COVID-19 mortality rates for the average U.S. American (11%) versus Asian Americans (6%) or African Americans (8%). Interestingly, Asian Americans perceived the risk of dying from COVID-19 as low for themselves (47%) but high for the average U.S. American (70%).

Regarding vaccine hesitancy within minoritized groups, more vaccine-hesitant individuals underestimated (vs. having accurate knowledge) the vaccine efficacy. They were also more likely to see COVID as a manufactured virus, as a hoax, that seasonal flu was deadlier than COVID, underestimate COVID mortality, and feel that the impact of COVID has been exaggerated. They were also more likely to have lower perceptions of dying from COVID than those of their respective racial and ethnic groups open to vaccination.

### 3.3. Racial/Ethnic Differences in Vaccine Hesitancy

Table 3 summarizes differences in vaccine hesitancy rates among the three racial/ethnic groups. Despite access to the vaccine, 28% of Black, 19% of Hispanic, and 8% of Asian American HCWs were vaccine-hesitant. Asian Americans were the most willing to accept and recommend the vaccine if hesitant (48%), while vaccine-hesitant Black participants were the least inclined to recommend the vaccination (27%). Asian American and Hispanic vaccine hesitancy were specific to the COVID-19 vaccine, whereas Black individuals reported lower uptake of other preventive immunizations (i.e., flu). Vaccine-hesitant Hispanic HCWs had the strongest negative attitudes toward the COVID-19 vaccine, with 60% reporting definitely or probably not being willing to be vaccinated.

### 3.4. Racial/Ethnic Differences in Predictors of Vaccine Hesitancy

The first model (Table 4a) assessed the direct effect (e.g., main effects) of the variables for predicting the outcome of vaccination status. The model provided a strong fit to the data (Likelihood ratio Chi sq = 969.85, df = 25, *p*-value < 0.001), and resulted in a 92.6% correct classification. Race/ethnicity, age, and education were significant predictors for the demographic variables. Overall, Black respondents were significantly less likely to be vaccinated, whereas Asian Americans were more likely to be vaccinated than White respondents in the total sample. Furthermore, older respondents were less likely to be vaccinated, as were respondents with higher levels of education. For the remainder of the variables (which were fitted with a backward stepwise removal), only occupation, clinic area, prior COVID-19 diagnosis, embrace of COVID-19 conspiracies, knowledge of COVID-19, and willingness to recommend the vaccine were significant in predicting vaccine hesitancy. Nurses and respiratory therapists were less likely to receive the vaccine. In contrast, respondents in an administrative role were more likely to receive the vaccine than the Allied Health Professionals reference group. In addition, respondents working in outpatient areas were more likely to be vaccinated than those working in the ICU (Intensive Care Unit) and non-ICU areas. Furthermore, respondents with a prior COVID-19 diagnosis and inadequate COVID-19 knowledge were less likely to be vaccinated. Those who had a lower acceptance of COVID-19 conspiracies and were willing to recommend the vaccine were more likely to be vaccinated. All other variables were excluded from the model as they did not add significance to the model.

Next, we assessed the retained variables from Model 1 and added race/ethnicity interaction effect. Most direct effects from Model 1 were estimated in a similar direction and magnitude in this second model, with a few exceptions. First, in model 2, as with model 1, Black respondents were less likely to be vaccinated, and Asian American respondents were more likely to be vaccinated. With the added interaction effects in model 2 (Table 4b), Hispanic respondents were estimated to be more likely to be vaccinated. This likelihood is due to the interaction effect between race/ethnicity and the clinic area of the respondent. This was the only interaction that was significant in the model. Specifically, Hispanic respondents in the ICU and E.R. were less likely to be vaccinated, but other Hispanic respondents (e.g., Non-ICU, Outpatient) were more likely to be vaccinated. This significant interaction also changed the ‘clinic work area’ direct effects. In model 2, only those who did not work at the time of the survey were less likely to be vaccinated than respondents who worked in outpatient areas. Finally, the direct effect for education level was reduced to be non-significant in this model. Model 2 retained all other significant effects from model 1 in model 2.

### 3.5. Racial/Ethnic Differences in Predictors of Vaccine Hesitancy

**Figure 1:**
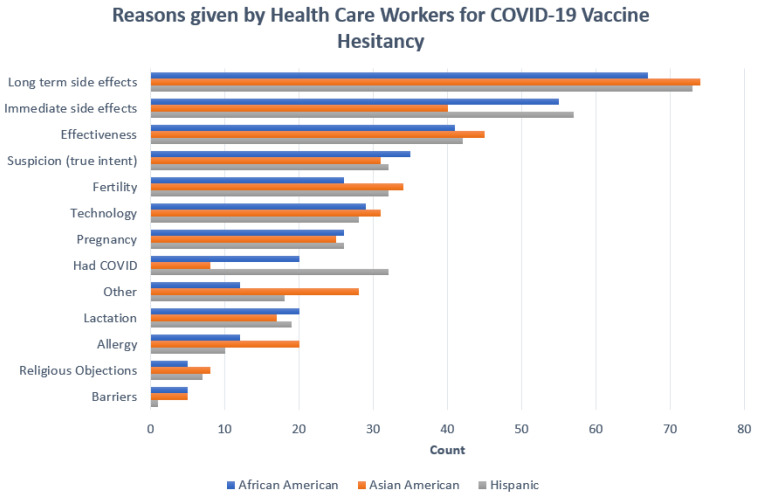
Ethnic variations in reasons given by health care workers for COVID-19 Vaccine Hesitancy.

The top 5 reasons (Figure 1) for vaccine hesitancy endorsed by 177 vaccine-hesitant minoritized HCWs were concerns about unrealized long-term side effects, immediate side effects, the vaccine’s effectiveness, suspicion, or governmental mistrust, and concerns about vaccine effects on fertility. 

A total of 34 of these participants gave additional reasons in their own words. It is essential to mention that religious objections to vaccination were among the least common reasons for hesitancy. While all HCWs were offered the vaccine, there might be racial differences in actual access (e.g., ability to take days off to cope with vaccine side effects) as evidenced by open-ended comments, such as “I want the hospital to cover my sick hours when I get sick from the vaccine” and “I want COVID so I can have two weeks off from work”, “we are OVER worked, out of ratio”. There were slight variations in reasons for vaccine hesitancy endorsed by the three minoritized groups. For instance, governmental mistrust and doubts regarding the vaccine development process were more prominent among Black respondents, supported by open-ended comments, such as “I don’t trust those really behind the scenes”, “approval process overly hasty”, and “not enough research is done and not enough time evaluating the vaccine”. Long-term side effects were the most critical reason for vaccine hesitancy among Asian Americans, as shown in open-ended comments “no long-term testing or documentation of adverse effects” and “not proven or tested to be proven safe and reliable”. Natural immunity was an important reason for vaccine hesitancy among Hispanic HCWs, shown in comments that “test subjects who were tested on hadn’t had COVID before so the approval isn’t for people who have had it before” and “not enough study on patients that have had COVID, receiving the vaccine”.

## 4. Discussion

The present study investigated the prevalence and factors associated with vaccine hesitancy among HCWs from minoritized communities at two hospitals in Southern California. The findings revealed that HCWs from minoritized communities are a heterogeneous group with varying degrees of vaccine hesitancy. Black HCWs were most hesitant about receiving the COVID-19 vaccine, while Asian Americans were least hesitant. There are various driving factors for vaccine hesitancy among specific racial and ethnic groups—the historical mistrust of the healthcare system among Black, the belief in physiological immunity coupled with misinformation among Hispanic, and the concern about long-term side effects among Asian American HCWs. These findings suggest the importance of recognizing and addressing the specific barriers to vaccination encountered by HCWs from minoritized communities to increase the saliency and efficacy of vaccine promotion messages [7]. By addressing the cultural, social, and historical forces that influence vaccine acceptance in this critical population, including those who may have relented and got vaccinated because of the mandate but may not be convinced otherwise, we could build the necessary trust and increase HCWs confidence in the COVID-19 vaccine going forward [18]. With the COVID-19 epidemic continuing to evolve, we will likely need our healthcare workforce to have more boosters. 

Therefore, rather than forcing the issue with requirements that have only resulted in early retirements and HCWs leaving the profession in California, vaccine hesitancy among HCWs of color needs to be examined through the prism of the social context (e.g., racism) in which the COVID-19 pandemic has unfolded. The country has been troubled by police brutality and moved by the Black Lives Matter protests and by White supremacist groups leading the attack on the U.S. Capitol on 6 January 2021 [19]. At the pandemic’s beginning, some elected leaders used offensive language, connecting the novel virus to the Asian community [20]. Such rhetoric caused waves of hatred and violence against Asian Americans in the U.S. [21]. The extreme distrust of government among minoritized communities resulted in concerns about COVID-19 vaccine safety and efficacy. Indeed, communities of color consistently ranked the federal government as the least reliable source of information about COVID-19 [22]. 

COVID-19-related misinformation poses a significant threat to communities of color, predominantly Hispanic individuals. A recent Voto Latino poll of Hispanics found that 78% believed COVID-19 misinformation significantly discouraged vaccine uptake [23]. Hispanics are particularly vulnerable to misinformation due to a greater reliance on social media and messaging platforms. They are over twice as likely to use messaging applications, such as WhatsApp and Telegram, as the general population [24]. The outsized use of these applications is conducive to spreading misinformation. At the same time, credible vaccine information and the science supporting it are not readily available in Spanish or, even if available, are not easily understood, given the complex issues involved. Better risk communication needs to present information in a more understandable and personally meaningful way.

Moreover, the term ‘vaccine hesitancy’ applied to HCWs of color likely also obscures a more complex set of realities, such as inequality in access [25]. Some of what is described as ‘hesitancy’ may have to do with a lack of or impeded access [26]. For instance, most ancillary staff and support personnel in both hospital systems are Black and Hispanic/Latino persons and likely have lower health literacy than highly educated health professionals. While vaccine promotion campaigns utilize emails and internal websites, many ancillary staff may have no access to a computer at home or at work. Vaccination sign-up processes have used computers and unique identifiers that some non-clinical frontline staff may lack. While all HCWs in this study have been offered the vaccine, some open comments suggest potential racial differences in actual access, such as one’s ability to take time off work to either wait for the vaccine or recover from its side effects.

Our study indicates the necessity of robust and tailored strategies to increase vaccinations among minoritized groups. Historical mistrust within the Black community, mainly attributable to the Tuskegee Syphilis Experiment and other horrific injustices, contributes to a reasonable lack of trust in the developmental process and long-term effects of the COVID-19 vaccines [27]. The divisive racial language of political leaders in that era also fueled mistrust [28]. Mistrust of the COVID-19 vaccine can be traced to the mistrust of the government that produced it and to the perception of racism and unequal treatment in healthcare systems that administer it [29]. Thus, vaccine skepticism among this group of HCWs can be interpreted as a coping response to centuries of injustices, ongoing police brutality, racial residential segregation, high poverty, and incarceration rates among Black people [30]. Vaccination campaigns tailored to reach this group of HCWs need to acknowledge systemic racism as a justifiable reason for vaccine hesitancy [31]. There needs to be an emphasis on placing trustworthy and representative healthcare champions to advocate for the vaccinations and decode the available scientific data attesting to their safety and efficacy. Several studies [32,33] have shown the success of utilizing culturally representative messengers as a part of vaccination outreach strategies within racially and ethnically minoritized communities. 

For Hispanics, it is crucial to address misinformation. Up-to-date scientific data needs to be translated and widely disseminated. These efforts may include social media campaigns to drive home accurate messages. Health education is also an essential issue for many minoritized communities, but data shows that it may be significant for Hispanics/Latinos. Routine health counseling and education on disease prevention and health promotion are necessary to reduce this population’s health disparities [34]. Lastly, Hispanics represent a large portion of U.S. minoritized groups and thus have been disproportionately affected by COVID-19. Culturally, some Hispanics tend to prefer holistic and natural remedies [35]. Hence, the notion of natural immunity may appeal to this group. Falling prey to the naturalistic fallacy, some may consider natural immunity superior to ‘artificial’ vaccine-induced immunity [36]. It is worth mentioning that prior COVID-19 infection offers a certain level of protection against subsequent infections and severe diseases [37,38,39]. However, a public health campaign tailored to this group needs to focus on the waning of natural protection after one year [40] and the benefits of a one-shot approach to prevent reinfection [41] and reduce the symptoms of long COVID-19 [42].

Asian Americans in the sample differed from other minoritized groups and would benefit from alternate prevention strategies. Campaigns highlighting the risks of COVID-19 to loved ones may be particularly compelling given the collectivist nature of many Asian Americans who had a larger number of older family members living with them in our sample. Collectivism means that the group’s needs are prioritized over that of the individual. Rightfully so, with a rapidly developed drug, people will also have concerns about the potential unknown long-term side effects. It will be helpful to provide easily understandable, clear, accurate, and timely data on the safety and outcomes of COVID-19 vaccines for Asian Americans. Thus, strategies for Asian Americans could include campaigns highlighting the risk to loved ones, having clear communication about expected side effects (including data on the odds of long-term side effects), and couched vaccination nudges in pro-social messaging.

While our study has many strengths, there are notable limitations. This study was conducted in the early months of COVID-19 vaccine availability, which to some extent is a limitation as vaccination mandates have since been issued, and the pandemic itself is a dynamic situation. For instance, with the emergence of new COVID-19 variants, vaccine efficacy wanes and case fatality rates fall, while the infectivity rate soars. Additionally, while we had a low response rate, it mirrors other surveys on the same topic systematically reviewed by Li et al. [43]. Given the taxing conditions during the data collection timeframe, having a sample of over 1131 HCWs from minoritized communities is substantial. The HCWs self-selected to participate, and the survey relies on self-report, which predisposes the results to possible bias. However, the vaccine hesitancy among this sample of HCWs is similar to the rates found in other studies worldwide [4,44], strengthening our findings. Finally, our cross-sectional study does not allow us to establish temporal causality or explore vaccination uptake. HCWs’ opinions on vaccination likely evolved. Hence, future surveys using validated instruments and relying on vaccination rates are needed to capture these changes.

## 5. Conclusions

This study found diversity in vaccine hesitancy among HCWs of color, with the highest rates among Black and Hispanic/Latino HCWs. We also observed various driving factors for vaccine hesitancy among specific racial and ethnic groups, including historical mistrust of healthcare, belief in natural immunity, misinformation, and concerns about long-term side effects of vaccination. These results suggest the need for targeted interventions that are mindful of the disparities in knowledge and access, differences between racial and ethnic groups, and prevailing mistrust of the medical system. Consequently, messaging should be tailored to specific groups, including trusted messengers, and should focus on particular concerns that shape attitudes toward COVID-19 vaccination.

## Figures and Tables

**Table 1:** Demographic Characteristics of the 1131 Health Care Workers of Color that responded to the survey, separated into three major ethnic groups. Results are reported as Count (Percent) in each column. Notable differences shaded in beige.

	Ethnicity	Total *(n = 1131)*	African American		Asian American		Hispanic	
			*Vaccinated* *(n = 89)*	*Hesitant* *(n = 34)*	*Vaccinated* *(n = 403)*	*Hesitant* *(n = 35)*	*Vaccinated* *(n = 462)*	*Hesitant* *(n = 108)*
**Gender**	*Male*	230 (20)	14 (16)	3 (9)	114 (28)	9 (26)	80 (17)	10 (9)
	*Female*	894 (79.5)	73 (82)	29 (85)	288 (72)	26 (74)	381 (83)	97 (90)
	*Non-binary*	7 (0.5)	2 (2)	2 (6)	1 (0.2)	0	1 (0.3)	1 (1)
**Year of Birth**	*1946–1964*	160 (14)	20 (21)	5 (15)	70 (17)	4 (11)	55 (12)	6 (6)
	*1965–1980*	386 (34)	33 (38)	14 (41)	149 (37)	11 (31)	150 (32)	29 (27)
	*1981–1996*	548 (48)	33 (39)	15 (44)	178 (44)	17 (50)	235 (51)	70 (65)
	*After 1996*	37 (3)	3 (2)	0	6 (2)	3 (8)	22 (5)	3 (3)
**Education**	*Some college*	200 (18)	11 (12)	4 (12)	21 (5)	1 (3)	137 (30)	26 (24)
	*Associate’s*	163 (14)	8 (9)	7 (20)	24 (6)	5 (14)	96 (21)	23 (21)
	*Bachelors*	349 (31)	24 (27)	9 (26)	138 (34)	17 (49)	124 (27)	37 (34)
	*Graduate*	160 (14)	23 (26)	10 (30)	47 (12)	5 (14)	58 (12)	17 (16)
	*Doctorate*	259 (23)	23 (26)	4 (12)	173 (43)	7 (20)	46 (10)	5 (5)
**Household Income**	*Less than 50k*	96 (8)	5 (5)	1 (3)	15 (4)	2 (6)	59 (13)	14 (13)
	*50–100k*	275 (24)	28 (32)	12 (35)	69 (17)	10 (29)	126 (27)	30 (28)
	*101–150k*	272 (24)	16 (22)	11 (32)	96 (24)	11 (31)	112 (24)	26 (24)
	*151–200k*	167 (15)	14 (16)	6 (18)	59 (15)	3 (8)	67 (16)	18 (17)
	*201–250k*	102 (9)	19 (8)	1 (3)	48 (12)	5 (14)	30 (6)	9 (8)
	*More than 250k*	129 (11)	10 (10)	2 (6)	84 (21)	0	29 (6)	4 (4)
	*Decline to state*	90 (8)	7 (6)	1 (3)	32 (7)	4 (11)	39 (8)	7 (6)
**Household Size**	*Alone*	100 (9)	14 (14)	3 (9)	39 (10)	2 (6)	35 (7)	5 (5)
	*Two*	303 (27)	27 (31)	12 (36)	102 (25)	6 (17)	136 (30)	20 (17)
	*Three*	229 (20)	20 (22)	8 (23)	88 (22)	6 (17)	80 (18)	27 (25)
	*Four*	272 (24)	16 (18)	8 (23)	101 (25)	14 (40)	102 (22)	31 (29)
	*More than four*	227 (20)	12 (13)	3 (9)	71 (18)	7 (20)	109 (23)	25 (23)
**Household member >65**	*Yes*	(18)	16 (18)	0	97 (24)	6 (17)	77 (17)	7 (6)
	*No*	928 (82)	73 (82)	34 (100)	306 (76)	29 (83)	385 (83)	101 (94)
**Political Affiliation**	*Democrat/lean Democrat*	558 (49)	59 (66)	17 (50)	202 (50)	5 (13)	241 (52)	34 (31)
	*Republican/lean Republican*	227 (20)	10 (11)	6 (18)	87 (21)	13 (37)	80 (18)	31 (29)
	*No lean*	346 (31)	20 (23)	11 (32)	114 (29)	17 (50)	141 (30)	43 (40)
**Occupation**	*Nurse*	388 (34)	29 (32)	13 (38)	126 (31)	20 (57)	162 (35)	38 (35)
	*Physician*	191 (17)	14 (16)	2 (6)	135 (34)	5 (14)	33 (7)	2 (2)
	*NP/PA*	27 (2)	5 (6)	0	10 (2)	0	11 (2)	1 (1)
	*Pharmacist*	32 (3)	1 (1)	0	27 (7)	1 (3)	2 (0.5)	1 (1)
	*Respiratory Therapist*	46 (4)	4 (5)	2 (6)	10 (2)	1 (3)	19 (4)	10 ()
	*Administration*	80 (7)	8 (9)	3 (9)	20 (5)	1 (3)	40 (9)	8 (7)
	*Ancillary Services*	367 (32)	28 (31)	14 (41)	75 (19)	7 (20)	195 (42)	48 (45)
**Clinical Area**	*ICU*	299 (26)	26 (29)	9 (26)	115 (29)	13 (37)	101 (22)	35 (32)
	*Non-ICU*	376 (33)	34 (38)	17 (50)	150 (37)	13 (37)	127 (27)	35 (32)
	*ER*	57 (5)	5 (6)	0	13 (3)	1 (3)	30 (7)	8 (7)
	*Outpatient*	376 (33)	23 (26)	8 (24)	115 (29)	8 (23)	193 (42)	29 (27)
	*Did not work*	23 (2)	1 (1)	0	10 (2)	0	11 (2)	1 (1)
**Medical Specialty**	*Adult critical care*	53 (5)	4 (5)	3 (9)	21 (5)	2 (6)	18 (4)	5 (5)
	*Adult gen med*	197 (17)	9 (10)	9 (26)	77 (19)	7 (20)	76 (17)	19 (17)
	*Adult subspecialty*	349 (31)	29 (32)	10 (29)	126 (31)	11 (31)	142 (30)	31 (29)
	*Peds critical care*	40 (3)	1 (1)	1 (3)	18 (4)	1 (3)	14 (3)	5 (5)
	*Peds gen med*	68 (6)	9 (10)	1 (3)	26 (6)	2 (6)	21 (5)	9 (8)
	*Peds subspecialty*	118 (10)	11 (12)	7 (20)	38 (9)	4 (11)	44 (10)	14 (13)
	*Surgery*	114 (10)	9 (10)	0	48 (12)	5 (14)	44 (10)	8 (7)
	*Emergency*	52 (5)	5 (6)	0	10 (2)	1 (3)	28 (6)	8 (7)
	*Non-medical*	140 (12)	6 (7)	9 (26)	39 (9)	2 (6)	75 (15)	9 (8)
**Contact with COVID patients**	*Frequent*	344 (30)	23 (26)	8 (24)	122 (30)	15 (43)	144 (31)	32 (30)
	*Intermittent*	358 (32)	31 (35)	12 (35)	148 (37)	8 (23)	119 (26)	40 (37)
	*No contact*	429 (38)	35 (39)	14 (41)	133 (33)	12 (34)	199 (43)	36 (33)
**Prior COVID diagnosis**	*Yes*	168 (15)	7 (8)	6 (18)	32 (8)	7 (19)	74 (16)	42 (39)
	*No*	963 (85)	82 (92)	28 (82)	371 (92)	28 (81)	388 (84)	66 (61)
**COVID financial impact**	*Yes*	520 (46)	47 (53)	14 (41)	174 (43)	16 (46)	211 (46)	58 (54)
	*No*	611 (54)	42 (47)	20 (59)	229 (57)	19 (54)	261 (54)	50 (46)
**COVID impact on family**	*Someone had COVID*	385 (34)	35 (39)	14 (41)	144 (36)	16 (46)	131 (28)	45 (42)
	*Someone was hospitalized*	197 (17)	16 (18)	8 (24)	70 (17)	7 (19)	77 (17)	19 (17)
	*Someone died or became disabled*	530 (47)	36 (41)	11 (32)	181 (45)	12 (35)	247 (53)	43 (40)
	*No one had COVID*	19 (2)	2 (2)	1 (3)	8 (2)	0	7 (2)	1 (1)
**Recent flu vaccination**	*Yes*	1027 (91)	78 (88)	21 (62)	384 (95)	21 (60)	433 (94)	90 (83)
	*No*	104 (9)	11 (12)	13 (38)	19 (5)	14 (40)	29 (6)	18 (17)

**Table 2:** Ethnic differences regarding various COVID-19 virus and COVID-19 vaccine beliefs amongst health care workers. Results are reported as Count (Percent) in each column. Notable differences shaded in beige.

Ethnicity	All Minorities *(n = 1131)*	African American		Asian American		Hispanic	
		*Vaccinated* *(n = 89)*	*Hesitant* *(n = 34)*	*Vaccinated* *(n = 403)*	*Hesitant* *(n = 35)*	*Vaccinated* *(n = 462)*	*Hesitant* *(n = 108)*
**Knowledge of COVID-19 vaccine efficacy**							
*Underestimate*	288 (25)	23 (26)	21 (62)	50 (12)	19 (54)	108 (23)	67 (62)
*Accurate*	843 (75)	66 (74)	13 (38)	353 (88)	16 (46)	354 (77)	41 (38)
**COVID-19 is a manmade virus**							
*Yes*	215 (19)	14 (16)	11 (33)	46 (11)	13 (37)	89 (19)	42 (39)
*Not sure*	254 (22)	24 (27)	10 (29)	78 (19)	10 (28)	104 (22)	28 (26)
*No*	662 (59)	51 (57)	13 (38)	279 (70)	12 (35)	269 (59)	38 (35)
COVID-19 is a hoax							
*Yes*	38 (3)	0 (0)	2 (6)	11 (4)	4 (11)	13 (3)	8 (7)
*Not sure*	59 (5)	2 (2)	4 (12)	16 (4)	3 (8)	25 (5)	9 (8)
*No*	1034 (92)	87 (98)	28 (82)	476 (92)	28 (81)	424 (92)	91 (85)
**The impact of COVID-19 is exaggerated**							
*Yes*	123 (11)	10 (11)	6 (18)	31 (7)	10 (28)	32 (7)	34 (31)
*Not sure*	69 (6)	3 (3)	2 (6)	16 (4)	2 (6)	33 (7)	13 (12)
*No*	939 (83)	76 (86)	26 (76)	356 (89)	23 (66)	397 (86)	61 (57)
**Seasonal flu is more contagious than COVID-19**							
*Yes*	313 (28)	17 (19)	7 (21)	106 (26)	10 (28)	146 (32)	27 (25)
*Not sure*	425 (37)	45 (51)	13 (38)	142 (35)	12 (35)	159 (34)	54 (50)
*No*	393 (35)	27 (30)	14 (41)	155 (39)	13 (37)	157 (34)	27 (25)
**Seasonal flu is deadlier than COVID-19**							
*Yes*	104 (9)	8 (9)	4 (12)	20 (5)	5 (14)	44 (9)	23 (21)
*Not sure*	315 (28)	19 (21)	13 (38)	96 (24)	15 (43)	134 (29)	38 (35)
*No*	712 (63)	62 (70)	17 (50)	287 (71)	15 (43)	284 (62)	47 (64)
**Estimated general population mortality from COVID-19**							
*Underestimate*	101 (9)	5 (6)	5 (15)	23 (6)	4 (11)	39 (9)	25 (23)
*Accurate*	319 (28)	22 (25)	6 (18)	114 (28)	12 (33)	131 (28)	34 (31)
*Overestimate*	413 (37)	29 (33)	11 (32)	162 (40)	17 (50)	167 (36)	27 (25)
*Not sure*	298 (26)	33 (36)	12 (35)	104 (26)	2 (6)	125 (27)	22 (20)
**Perceived self-likelihood of dying from COVID-19**							
*Low*	383 (34)	16 (18)	15 (44)	192 (48)	14 (40)	101 (22)	45 (42)
*Average*	197 (17)	15 (17)	5 (15)	35 (8)	6 (17)	113 (24)	23 (21)
*High*	250 (22)	22 (25)	8 (23)	78 (19)	9 (26)	115 (25)	18 (17)
*Don’t know*	304 (27)	36 (40)	6 (18)	101 (25)	6 (17)	133 (29)	22 (20)

**Table 3:** Ethnic differences regarding COVID-19 vaccine hesitancy amongst health care workers. Results are reported as Count (Percent) in each column. Notable differences shaded in beige. * Denominator denotes number of individuals in cohort that received at least one COVID-19 vaccination.

Ethnicity	All Minorities *(n = 1131)*	African American		Asian American		Hispanic	
		*Vaccinated* *(n = 89)*	*Hesitant* *(n = 34)*	*Vaccinated* *(n = 403)*	*Hesitant* *(n = 35)*	*Vaccinated* *(n = 462)*	*Hesitant* *(n = 108)*
**Willingness to receive COVID-19 vaccine**							
*No*			18 (52)		12 (34)		63 (59)
*Not sure*			9 (26)		7 (20)		23 (21)
*Yes*			7 (21)		16 (46)		32 (20)
**Willingness to recommend COVID-19 vaccine**							
*No*	68 (6)	1 (1)	14 (41)	1 (0.2)	8 (22)	2 (0.5)	42 (39)
*Not sure*	87 (8)	1 (1)	11 (32)	11 (3)	10 (29)	18 (4)	36 (33)
*Yes*	976 (86)	87 (98)	9 (27)	391 (97)	17 (48)	442 (96)	30 (28)
Willingness to receive third vaccination dose *							
*No*		1 (1)		14 (3)		10 (2)	
*Not sure*		16 (18)		27 (7)		48 (11)	
*Yes*		72 (81)		362 (90)		404 (87)	

**Table 4:** (**a**): Predictors of Vaccine Hesitancy using a direct effects model. Odds ratio < 1 indicates group less likely to be vaccinated, while Odds ratio > 1 indicates group more likely to be vaccinated. Significant differences shaded in beige (less likely to be vaccinated) and blue (more likely to vaccinate). (**b**): Predictors of Vaccine Hesitancy using a direct effects model with ethnicity interaction effects. Only interaction effects of Hispanics with clinical area shown as that was the only interaction significant in Model 2. Odds ratio < 1 indicates group less likely to be vaccinated, while Odds ratio > 1 indicates group more likely to be vaccinated. Significant differences shaded in beige (less likely to vaccinate) and blue (more likely to be vaccinated).

(a)						
	MODEL 1			MODEL 2		
	*Direct Effects Backward Stepwise*			*Direct Effect Only*		
	*Adjusted Odds Ratio*	*95% CI*	*p-Value*	*Adjusted Odds Ratio*	*95% CI*	*p-Value*
**Ethnicity**						
*African American*	0.555	0.288–1.072	0.08	0.561	0.289–1.087	0.09
*Asian American*	1.853	1.098–3.127	0.02	1.871	1.106–3.165	0.02
*Hispanic*	1.271	0.825–1.959	0.28	2.721	1.226–6.035	0.01
**Male**	1.031	0.646–1.643	0.89	1.053	0.660–1.679	0.83
**Age**	0.659	0.520–0.836	<0.001	0.628	0.493–0.802	<0.001
**Income**	0.989	0.843–1.159	0.89	0.993	0.846–1.166	0.93
**Education**	0.841	0.691–1.024	0.08	0.87	0.846–1.057	0.16
**Household Size**	0.934	0.812–1.074	0.34	0.933	0.809–1.074	0.33
**Chronic Illness**	1.101	0.747–1.624	0.63	1.105	0.748–1.632	0.62
**Occupation [REFERENCE = Patient Care Assistant]**						
*Nurse*	0.648	0.424–0.992	0.05	0.634	0.413–0.974	0.04
*Physician, Attending*	1.94	0.647–5.820	0.24	1.832	0.606–5.543	0.28
*Physician, Resident*	1.687	0.497–5.725	0.40	1.553	0.452–5.543	0.49
*Physician, Fellow*	1.163	0.124–10.988	0.90	0.971	0.103–9.164	0.98
*Nurse Practitioner or Physician Assistant*	1.173	0.328–4.196	0.81	1.085	0.306–3.843	0.90
*Pharmacist*	0.562	0.178–1.774	0.33	0.481	0.152–1.519	0.21
*Respiratory Therapist*	0.481	0.212–1.088	0.08	0.498	0.219–1.132	0.10
*Administration*	3.146	1.321–7.493	0.01	2.914	1.222–6.948	0.02
**Clinical Area [REFERENCE = Outpatient]**						
*ICU*	0.608	0.362–1.021	0.06	0.926	0.509–1.683	0.80
*Non-ICU*	0.574	0.359–0.917	0.02	0.722	0.417–1.251	0.25
*ER*	0.806	0.394–1.648	0.55	1.237	0.525–2.916	0.63
*Didn’t Work*	0.275	0.087–0.865	0.03	0.231	0.064–0.835	0.03
**Prior COVID Diagnosis**	0.182	0.121–0.273	<0.001	0.165	0.109–0.251	<0.001
**Low acceptance of COVID conspiracies**	1.391	1.099–1.762	0.006	1.407	1.109–1.785	0.005
**Inadequate COVID-19 knowledge**	0.407	0.275–0.603	<0.001	0.393	0.264–0.585	<0.001
**Willingness to recommend vaccine**	7.147	5.787–8.826	<0.001	7.302	5.892–9.048	<0.001
**(b)**						
**MODEL 2** ***Interaction Effects for Hispanics***						
	***Adjusted Odds Ratio***		***95% CI***		***p-Value***	
**Clinical Area [REFERENCE = Outpatient]**						
*ICU*	**0.230**		**0.80–0.664**		**0.007**	
*Non-ICU*	0.516		0.192–1.389		0.19	
*ER*	**0.267**		**0.059–1.213**		**0.09**	
*Didn’t Work*	3.281		0.166–64.992		0.44	

## Data Availability

The data that support the findings of this study are not openly available due to institutional policies and are available from the corresponding author upon reasonable request in a controlled access.
